# Evidence for a coordinate role of CD14+ antigen-presenting cells and regulatory T cells in conditioning the microenvironment of metastatic lymph nodes from patients with cervical cancer

**DOI:** 10.1186/2051-1426-2-S3-P211

**Published:** 2014-11-06

**Authors:** Marijne Heeren, Bas Koster, Sanne Samuels, Debbie Ferns, Dafni Chondronasiou, Gemma Kenter, Ekaterina S Jordanova, Tanja D de Gruijl

**Affiliations:** 1 Obstetrics & Gynecology, VU University Medical Center, Amsterdam, The Netherlands; 2 Medical Oncology, VU University Medical Center, Amsterdam, The Netherlands; 3 Gynecology, The Netherlands Cancer Institute, Amsterdam, The Netherlands

## 

A better understanding of the microenvironment in relation to lymph node metastasis is essential for the development of effective immunotherapeutic strategies against cervical cancer.

In the present study, we investigated the microenvironment of tumor-draining lymph nodes of cervical cancer patients, by comprehensive flow cytometry-based phenotyping and enumeration of immune-cell subsets in tumor-negative (LN-, n = 20) versus tumor-positive lymph nodes (LN+, n = 8), and by the study of cytokine release profiles (n = 4 for both LN- and LN+).

We found significantly lower CD4^+ ^and higher CD8^+ ^T-cell frequencies in LN+ samples, accompanied by increased surface levels of activation (HLA-DR and ICOS) and inhibitory markers (PD-1 and CTLA-4). Furthermore, in LN+ we found increased rates of a potentially regulatory antigen-presenting cell (APC) subset (CD11c^hi^CD14^+^PD-L1^+^) and of myeloid-derived suppressor cell (MDSC) subsets, which in the case of the former correlated significantly with elevated frequencies of FoxP3^+ ^Tregs. After *in vitro *stimulation with different TLR ligands (PGN; Poly-IC; R848), we observed higher production levels of IL-6, IL-10 and TNFα but lower levels of IFNγ in LN+.

We conclude that, despite increased T-cell differentiation and activation, a striking switch to a profound immune suppressive microenvironment in LN+ of cervical cancer patients will enable immune escape. Our data point to the CD14^+^PD-L1^+ ^APC/Treg axis as a particularly attractive and relevant therapeutic target to specifically tackle microenvironmental immune suppression and thus enhance the efficacy of immunotherapy in patients with metastasized cervical cancer.

